# Suppression of NLRP3 Inflammasome by Dihydroarteannuin *via* the HIF‐1α and JAK3/STAT3 Signaling Pathway Contributes to Attenuation of Collagen-Induced Arthritis in Mice

**DOI:** 10.3389/fphar.2022.884881

**Published:** 2022-04-27

**Authors:** Mingying Zhang, Danbin Wu, Jia Xu, Lijuan Liu, Wei Jiao, Jiahui Yu, Guangxing Chen

**Affiliations:** ^1^ Department of Rheumatology, The First Affiliated Hospital of Guangzhou University of Chinese Medicine, Guangzhou, China; ^2^ First Clinical Medical School, Guangzhou University of Chinese Medicine, Guangzhou, China; ^3^ Baiyun Hospital of The First Affiliated Hospital of Guangzhou University of Chinese Medicine, Guangzhou, China

**Keywords:** dihydroarteannuin, rheumatoid arthritis, traditional Chinese medicine, Jak3/Stat3, NLRP3, CIA mice, HIF- 1 α

## Abstract

Dihydroarteannuin (DHA), the primary element of artemisinin extracted from the traditional Chinese herb *Artemisia annua* L., has been used in malaria treatment for a long time. Recently, many studies have indicated that DHA also exhibits potent anti-rheumatoid arthritis (RA) activity. In this study, collagen-induced arthritis (CIA) in DBA/1J mice and inflammatory model in THP-1 cells were established to evaluate the modulatory effects of DHA on joint destruction and to explore the underlying mechanisms. Our results showed that DHA decreased the serum levels of IL-1β and IL-6, alleviated paw oedema, and reduced bone destruction in DBA/1J mice with CIA. Further exploration with the inflammatory model in THP-1 cells indicated that DHA reduced the protein expression of hypoxia‐inducible factor (HIF)‐1α and the phosphorylation in Janus kinase (JAK) 3 and signal transducer and activator of transcription (STAT) 3 protein, which resulted in a decrease in NOD-like receptor protein (NLRP) 3 expression and interleukin (IL)-1β release. Consequentially, the inflammatory activation in THP-1 cells was inhibited. Therefore, we concluded that DHA efficiently alleviated the inflammation and arthritic symptoms in CIA mice and downregulated inflammation in part by inhibiting NLRP3 expression *via* the HIF‐1α and JAK3/STAT3 signaling pathway. Thus, DHA may be considered as a potential therapeutic agent in RA treatment.

## Introduction

Rheumatoid arthritis (RA) is an autoimmune inflammatory disease characterized by chronic synovitis and progressive cartilage and bone destruction and ultimately leads to joint damage or even irreversible disability. Relief of synovial inflammation has long been the key to RA treatment, as persistent synovial inflammation is the dominant pathological manifestation of RA (Scott et al., 2010). Disease-modifying antirheumatic drugs (DMARDs), such as methotrexate (MTX), leflunomide, and sulfasalazine, are able to interfere with the synovial inflammatory process. However, owing to their toxicity, these may not always be ideal therapeutic agents. Biological agents such as tumor necrosis factor (TNF) inhibitors and interleukin (IL)-6 receptor inhibitors can be utilized when arthritis is uncontrolled or toxic effects arise with DMARDs. However, infections and high costs often restrict prescription of biological agents (Singh et al., 2015). Therefore, there continues to be an urgent need to develop improved and affordable therapeutic agents for RA.

Dihydroarteannuin (DHA) is an active metabolite of artemisinin and its derivatives, which has been demonstrated to be an effective antimalarial drug with low toxicity (Tu, 2011; Dai et al., 2021). Numerous hints were accumulated during the past years that DHA exhibited powerful anti-inflammatory properties and might also be of therapeutic interest for some autoimmune inflammatory disease (Efferth, 2017). In 2006, data from Tu Youyou’s group showed that DHA inhibited the secretion of TNF-α by blocking the nuclear factor (NF)-κB signaling pathway, which may be beneficial for the treatment of systemic lupus erythematosus related nephritis (Li et al., 2006). Besides, DC32, a DHA derivative, significantly suppressed the CIA *via* the Nrf2-p62-Keap1 feedback loop (Fan et al., 2018a) and inhibiting lymphocytic infiltration through downregulating the expression and transcription of IL-6 (Fan et al., 2018b). Another study in ulcerative colitis showed that DHA impeded inflammation by inhibiting the IL-6/signal transducer and activation of the transcription (STAT) 3 signaling pathway (Jiang et al., 2021a). STAT3 is one of the major signal transducers of JAKs and can be phosphorylated by JAK3. The elevated expression and phosphorylation of STAT3 lead to the persistence of synovial inflammation in RA (Gao et al., 2015). Therefore, STAT3 has provided a new potential therapeutic strategy to prevent synovitis in RA, and we wondered whether DHA could alleviate synovitis in RA by inhibiting STAT3 phosphorylation.

Previous studies showed that hypoxia‐inducible factor (HIF)‐1α, the most direct regulatory factor of the adaptive response to alterations in oxygen tension (Muz et al., 2009), took part in the phosphorylation of STAT3. Synovial tissue in RA patients is hypoxic (Sivakumar et al., 2008), which leads to an accumulation of HIF-1α in there. Ultimately, HIF-1α activated the JAK/STAT signaling pathway and increases STAT3 phosphorylation (Zhu et al., 2019). Then, the phosphorylation of STAT3 upregulates NOD-like receptor protein (NLRP) 3 inflammasome expression by enhancing acetylation of histones H3 and H4 on the NLRP3 promoter (Liu et al., 2018; Zhu et al., 2021). Besides, NLRP3 inflammasome is a multiprotein complex that is involved in IL-1β maturation and plays an important part in the inflammatory response (He et al., 2016).

On the basis of the above information, we assumed that DHA reduced inflammatory response in RA by inhibiting NLRP3 expression *via* the HIF‐1α and JAK3/STAT3 signaling pathway. In the present study, the role of DHA in modulating the inflammation and consequently protecting against bone erosion in CIA mice was examined.

## Materials and Methods

### Materials

DHA (Cat. No. S2290) was purchased from Selleck Chemicals. Lipopolysaccharide (LPS, Cat. No. L2630) and phorbol myristate acetate (PMA, Cat. No. P8139) were purchased from Sigma. Complete Freund’s adjuvant (CFA, Cat. No. 7001), incomplete Freund’s adjuvant (IFA, Cat. No. 7002), and bovine type II collagen (Cat. No. 20021) were purchased from Chondrex. Roswell Park Memorial Institute (RPMI) 1640 medium and fetal bovine serum (FBS) were obtained from Gibco. Cell Counting Kit 8 (CCK-8, Cat. No. HY-K0301) was purchased from Med Chem Express. RIPA buffer was purchased from Solarbio Life Sciences (Cat. No. R0020). BCA detection kit was purchased from Thermo Fisher Scientific (Cat. No. 23225). The primary antibodies against STAT3 (Cat. No. 9139), Phospho-STAT3 (Cat. No. 9145), JAK3 (Cat. No. 8863), Phospho-JAK3 (Cat. No. 5031), and GAPDH (Cat. No. 2858) were purchased from Cell Signaling Technology. The primary antibodies against NLRP3 (Cat. No. PA5-79740) were purchased from Invitrogen. The primary antibodies against HIF‐1α (Cat. No. sc-13515) were purchased from Santa Cruze. Goat Anti-Rabbit IgG (H + L) HRP (Cat. No. 98164) and Horse Anti-Mouse IgG (H + L) HRP (Cat. No. 91196) were purchased from Cell Signaling Technology.

### Animal Husbandry

Eight-week-old male DBA/1J mice (Beijing HFK Bioscience Co., Ltd., Beijing, China) were used for this experiment. All mice were raised in standard cages under a controlled light/dark cycle of 12 h at 22 ± 2°C and a humidity of 45 ± 5%. All the experimental studies were strictly in accordance with Guangzhou University of Chinese Medicine Animal Ethics Committee guidelines for the rational use of animals.

### Induction of Collagen-Induced Arthritis

After a week of acclimatization, all mice were randomly divided into two groups: control group (n = 6) and arthritis-induced group (n = 18). Bovine type II collagen was fully emulsified with an equal volume of CFA. 0.1 ml of the emulsion was injected intradermally at approximately 1.5 cm distal to the base of the tail. A booster injection of 0.1 ml emulsion of Bovine type II collagen and IFA was administered intradermally into the back on day 21. Mice in the control group were injected with normal saline at the same location and frequency as the arthritis-induced group.

### Drug Administration

From day 21 after the second immunization, mice in the arthritis-induced group were randomly divided into three groups (n = 6 per group): model group, DHA group, and MTX group. 20 mg/kg of DHA was orally given for mice in the DHA group every day. 2 mg/kg of MTX was orally given for mice in the MTX group every 3 days as the positive control. DHA and MTX were dissolved in corn oil. Mice in the control and model groups were orally given an equal volume of corn oil in parallel.

### Assessment of CIA

From day 21 after the second immunization, clinical arthritis scores were determined every week using a scoring system of 0–4 for each limb: 0, normal; 1, definite redness and swelling of the ankle or one digit; 2, two joints involved; 3, more than two joints involved; and 4, severe arthritis of the entire paw and all digits. Meanwhile, swelling of hind paws was measured using digital calipers, and body weight was recorded during the experiment.

### Micro-CT Analysis

At the end of the experiment, the right hind limb of each mouse was collected and scanned using the Skyscan 1,176 Micro-CT Imaging System (Skyscan, Kontich, Belgium). The scanning was carried out using the following settings: voltage, 80 kV; source current, 88μA; pixel size 4 μm. Two- and three-dimensional images were generated using Data-viewer and CTvol softwares (Bruker micro-CT, Kontich, Belgium), respectively. The bone mineral density (BMD) and bone volume/tissue volume (BV/TV) were evaluated using CT Analyzer program (Bruker micro-CT, Kontich, Belgium).

### Cell Culture

THP-1 cells, a pro-monocytic cell line, were obtained from American Type Culture Collection (ATCC) and cultivated in RPMI-1640 media supplemented with 10% FBS. THP-1 cells were cultured at a density of 5×10^5^ cells/ml in a humidified chamber with 5% CO_2_ at 37°C. In all experiments, THP-1 cells were cultured in 6-well plates treated with 100 ng/ml PMA for 24 h to transform them into adherent macrophages. Then, THP-1–derived macrophages were pretreated with different concentrations of DHA for 2 h, followed by stimulation of 100 ng/ml LPS for 24 h to establish the *in vitro* inflammation model.

### Cell Viability Assay

THP-1–derived macrophages were incubated with different concentrations of DHA for 24 h. Then, CCK-8 solution was added, and the cells were incubated in a humidified chamber with 5% CO_2_ at 37°C for 1–4 h. The absorbance at 490 nm was measured using a microplate reader for the detection of cell viability.

### Cytokine Assay

At the end of the experiment, blood samples of mice were collected. Serums were harvested by spinning of whole blood at 3,000 rpm for 15 min at 4°C. Serum levels of IL-1β (Cat. No. E08054m, CUSABIO) and IL-6 (Cat. No. E04639m, CUSABIO) were determined using ELISA kits. After DHA and LPS treatment for 24 h, the supernatant was collected from cell cultured medium and its IL-1β level was measured using ELISA kit (Cat. No. E08053h, CUSABIO).

### Western Blot

After being treated with DHA and LPS, cells were harvested and lysed by RIPA buffer for the total protein collection. After the protein concentration was evaluated using a BCA detection kit, the protein was mixed with loading buffer, and denaturation was carried out by heating under 97°C for 7 min. The target protein was separated by SDS-PAGE, transferred to a PVDF membrane, sealed with 5% skimmed milk at room temperature for 2 h, and incubated with one of the following primary antibodies at 4°C overnight: rabbit anti-STAT3, rabbit anti-Phospho-STAT3, rabbit anti-JAK3, rabbit anti-Phospho-JAK3, rabbit anti-NLRP3, mouse anti-HIF-1α, and rabbit anti-GAPDH. Then, the membranes were incubated with the corresponding secondary antibodies (goat anti-rabbit IgG or horse anti-mouse IgG) for 2 h following visualization through the Enhanced Chemiluminescence (ECL) method. Last, the images were recorded in a gel documentation system (ChemiDoc™ MP, Bio-Rad, CA).

### Statistical Analysis

Statistical data were shown as means ± SD. SPSS software (Version 18.0) was used to analyze data, while GraphPad Prism software (version 7.0) was used to draw graphs. One-way ANOVA with Newman-Keuls multiple comparisons test was applied for normal distribution and homogeneous variance. Dunnett’ T3 test was used for non-normal distribution. *p* < 0.05 indicates statistical significance.

## Result

### DHA Improved Body Weight in CIA Mice

**FIGURE 1:**
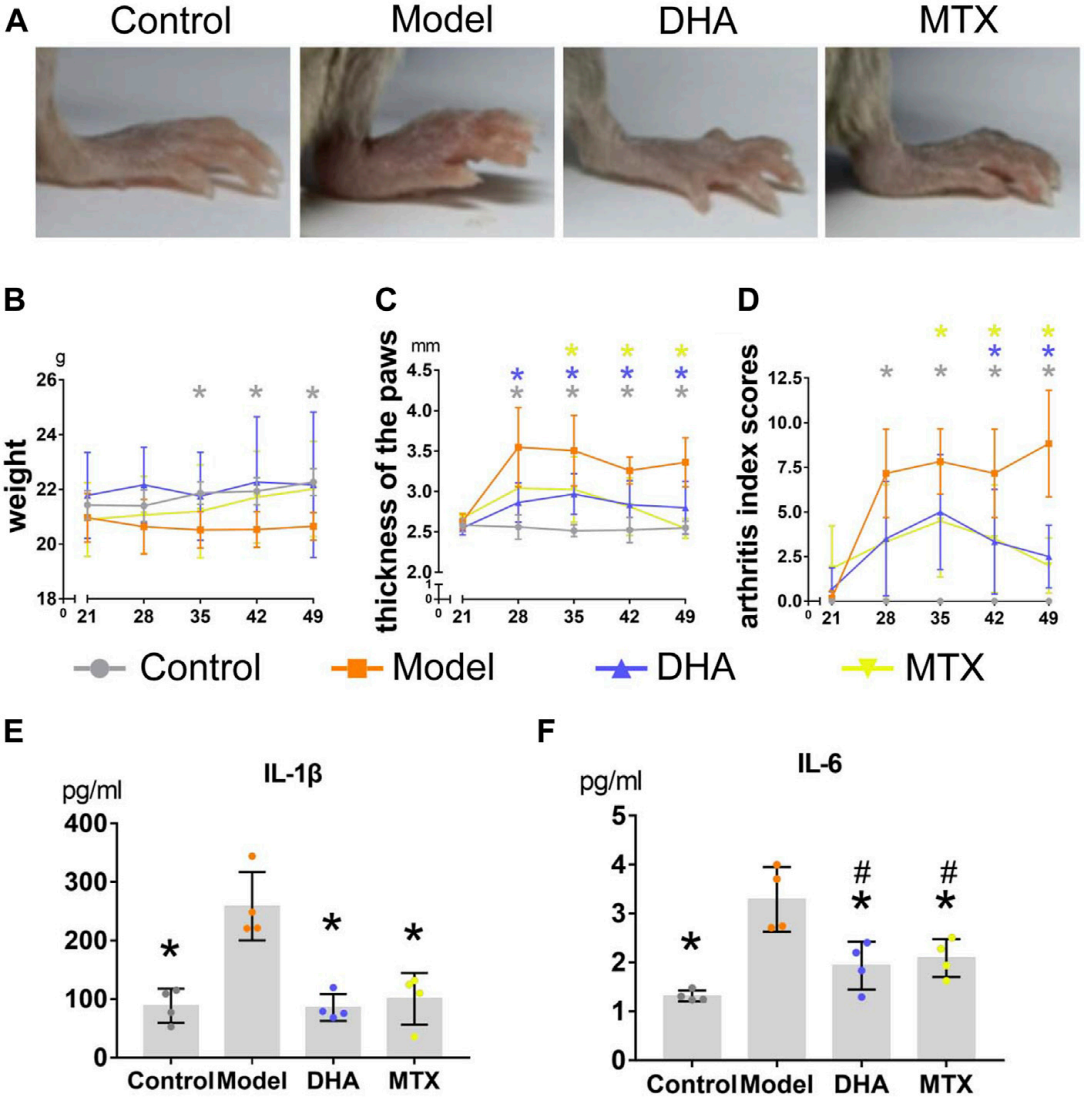
DHA treatment mitigated clinical symptoms in CIA mice. **(A)** Paws oedema were photographed. **(B)** Body weight were recorded weekly during the experiment. **(C)** The thickness of hind paws was measured weekly by digital calipers. **(D)** The clinical arthritis index scores were determined every week using a scoring system of 0–4 for each limb. **(E,F)** Serum levels of IL-1β and IL-6 were determined using ELISA kits. Data are presented as the mean ± SD. *p < 0.05, **p < 0.001 *vs.* the model group. ^#^ *p* < 0.05 *vs.* the control group.

Each mouse was weighed weekly to assess whether the treatment of DHA (20 mg/kg·d) or MTX (2 mg/kg·3 d) influenced weight gain in CIA mice. As illustrated in Figure 1B, the body weight increased smoothly throughout the experiment in the control group. When comparing the control group with the model group, the weight in the latter group had declined significantly between day 35 and day 49. Besides, there was no difference in body weight among the control, DHA, and MTX groups over the course of the experiment.

### DHA Alleviated Paw Oedema in CIA Mice

After the secondary immunization (day 21), paw oedema appeared dramatically in mice with CIA (Figure 1A). Hind paw oedema was measured weekly using digital calipers (Figure 1C). When compared to the control group, the thickness of hind paws in the model group was significantly increased between day 28 and day 49 (*p <* 0.05). Mice in the DHA group showed significant reduction in paw oedema between day 28 and day 49 compared with mice in the model group (*p <* 0.05). Mice in the MTX group also showed significant reduction in paw oedema between day 35 and day 49 compared with mice in the model group (*p <* 0.05). Additionally, a significant reduction in arthritis index scores also occurred in CIA mice with DHA or MTX treatment from day 35 and day 49, when compared to the model group (Figure 1D, *p <* 0.05). There was no significant difference between the DHA and MTX groups when it came to paw oedema or arthritis index scores.

### DHA Ameliorated the Bone Loss in CIA Mice

**FIGURE 2:**
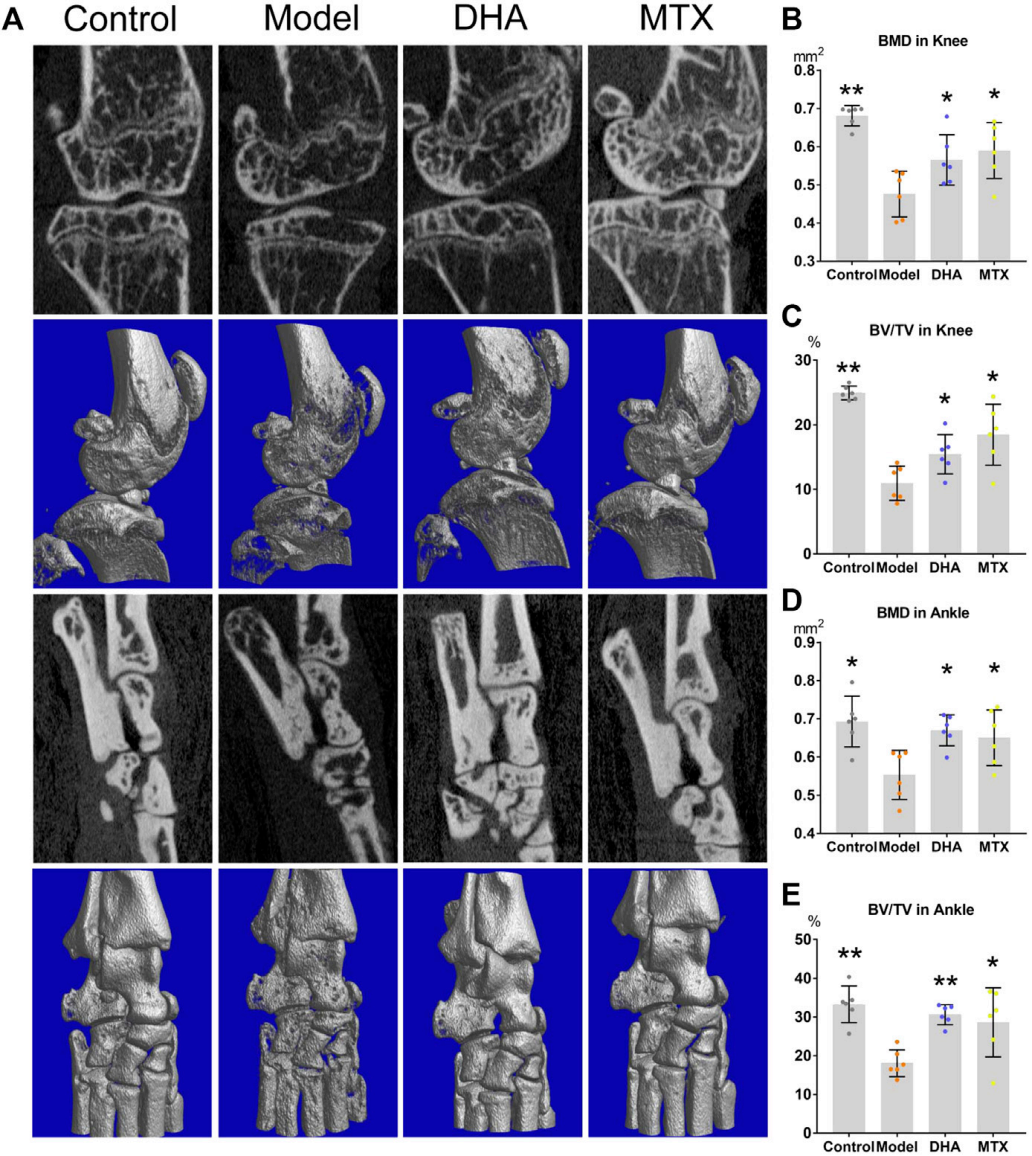
DHA treatment mitigated CIA bone erosion. **(A)** The right hind ankle and knee of each mouse were collected and scanned using the Skyscan 1,176 Micro-CT Imaging System. **(B–E)** The bone mineral density (BMD) and bone volume/tissue volume (BV/TV) were calculated. *p < 0.05, **p < 0.001 vs. the model group.

For measuring of bone mineral density (BMD) and bone volume/tissue volume (BV/TV, %), the articular structure of hind knees and ankles were scanned by micro-CT (Figure 2A). As showed by Figures 2B–E, in comparison to the control group, the BMD and BV/TV in the knees and ankles of the model group were significantly decreased (knee: 0.68 ± 0.03 *vs.* 0.48 ± 0.06 mm^2^, *p <* 0.001; 24.93 ± 1.05 *vs.* 10.95 ± 2.65%, *p <* 0.001; ankle: 0.69 ± 0.07 *vs.* 0.55 ± 0.06 mm^2,^, *p <* 0.05; 33.27 ± 4.73 *vs.* 18.06 ± 3.47%, *p <* 0.001). DHA or MTX treatment ameliorated the bone loss in CIA mice, as the BMD (knee: 0.57 ± 0.07 mm^2^, 0.59 ± 0.07 mm^2^, *vs.* model group, *p <* 0.05; ankle: 0.67 ± 0.04 mm (Singh et al., 2015), 0.65 ± 0.07 mm^2^, *vs.* model group, *p <* 0.05) and BV/TV (knee:15.46 ± 3.05%, 18.47 ± 4.72%, *vs.* model group, *p <* 0.05; ankle: 30.60 ± 2.59%, 28.26 ± 8.92%, *vs.* model group, *p <* 0.001, *p <* 0.05, respectively) in these two groups were significantly higher than those in the model group. No meaningful difference between the DHA and MTX groups was observed in BMD or BV/TV.

### DHA Decreased the IL-1β and IL-6 Levels in Serum of CIA Mice

Serum was harvested at the end of the experiment and the circulating levels of IL-1β and IL-6 were detected by ELISA. As shown in Figures 1E,F, there were higher circulating levels of IL-1β and IL-6 in the model group than in the control group (IL-1β: 258.73 ± 58.34 *vs.* 88.64 ± 29.14 pg/ml, *p <* 0.05; IL-6: 3.29 ± 0.66 *vs.* 1.32 ± 0.11 pg/ml, *p <* 0.05). Both the DHA and MTX groups had a much lower level of IL-1β and IL-6 than the model group (IL-1β: 85.80 ± 23.01, 100.68 ± 44.18 pg/ml, *vs.* model group, *p <* 0.05; IL-6: 1.93 ± 0.49, 2.08 ± 0.39 pg/ml, *vs.* model group, *p <* 0.05). No significant difference between the DHA and MTX groups was observed in circulating levels of IL-1β or IL-6.

### Cytotoxicity Effect of DHA in THP-1–Derived Macrophages

**FIGURE 3:**
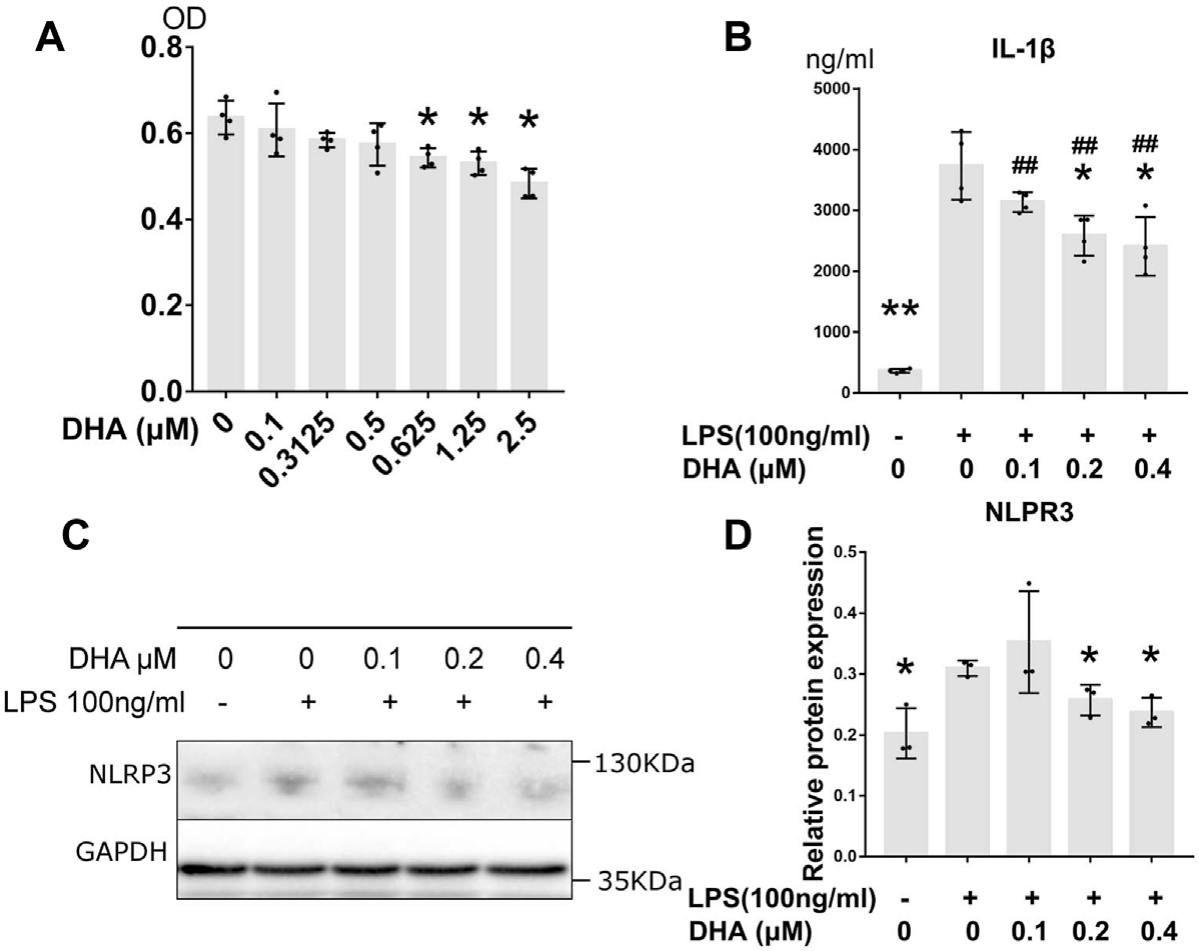
DHA treatment inhibited inflammatory response in macrophages with LPS stimulation. **(A)** The cytotoxicity of DHA at concentrations of 0.1–2.5 μM in THP-1–derived macrophages were detected by CCK-8 assay. **(B)** The IL-1β release at 24 h after incubation with or without LPS (100 ng/ml) and DHA (0.1, 0.2, and 0.4 μM). **(C,D)** The NLRP3 expression at 24 h after incubation with or without LPS (100 ng/ml) and DHA (0.1, 0.2, and 0.4 μM). *p < 0.05, **p < 0.001 vs. the model group. #*p* < 0.05, ##*p* < 0.001 vs. the control group.

The cytotoxicity of DHA at concentrations of 0.1–2.5 μM in THP-1–derived macrophages were detected by CCK-8 assay (Figure 3A). The result showed that no cytotoxicity was observed at doses of DHA up to 0.5 μM. Three lower doses of DHA, 0.1, 0.2, and 0.4 μM, were used in the subsequent studies.

### DHA Reduced the LPS-Induced IL-1β Release *via* NLPR3 Inhibition

Effects of DHA (0.1, 0.2, and 0.4 μM) on LPS-mediated IL-1β release were evaluated by ELISA (Figure 3B). After LPS triggering, the production of IL-1β dramatically increased (*p <* 0.001), whereas DHA was able to partially attenuate the secretion of IL-1β at the doses of 0.2 and 0.4 μM. The maturation of IL-1β is mainly mediated by NLRP3 inflammasome. Subsequent Western blotting with anti-NLRP3 demonstrated that LPS increased NLRP3 levels in these cells. However, DHA at the doses of 0.2 and 0.4 μM decreased the NLRP3 levels in macrophages under LPS triggering (Figures 3C,D).

### DHA Reduced LPS-Induced HIF‐1α Expression and JAK3/STAT3 Signaling

**FIGURE 4:**
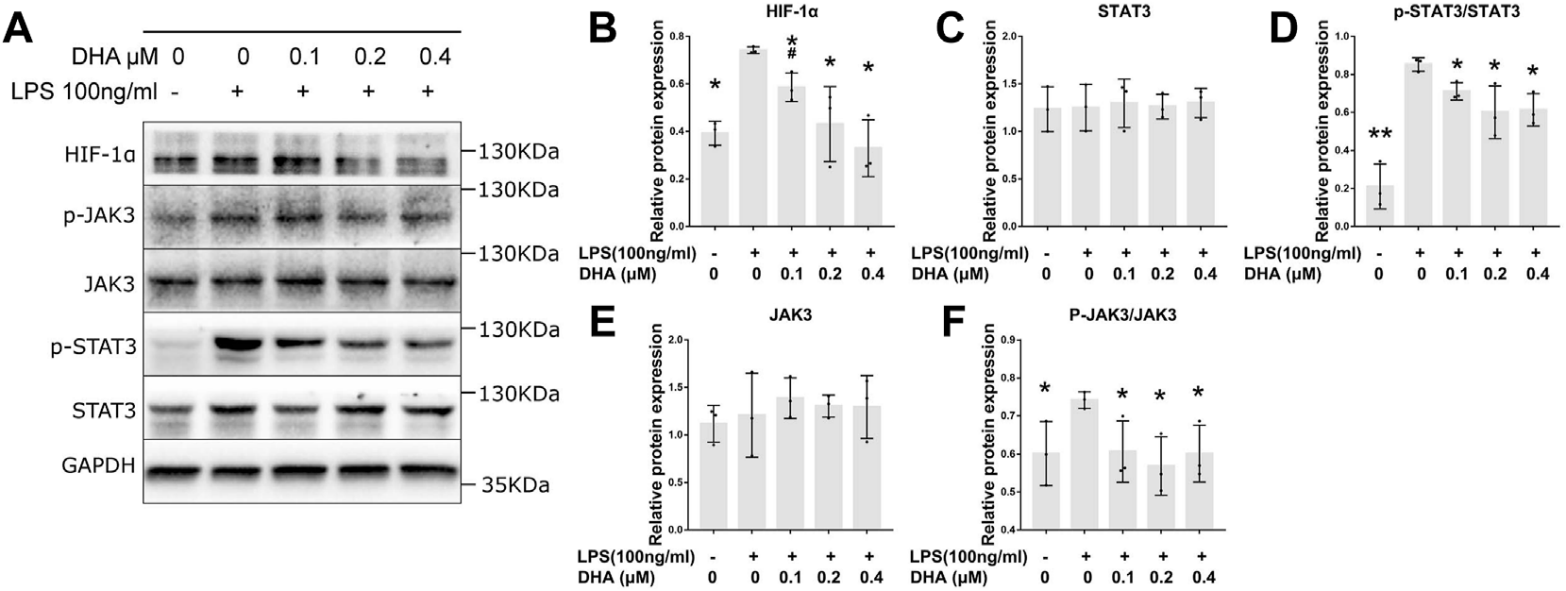
DHA treatment inhibited the HIF‐1α and JAK3/STAT3 signaling pathway in macrophages with LPS stimulation. **(A–F)** The expression of HIF‐1α, JAK3, p-JAK3, STAT3, and p-STAT3 at 24 h after incubation with or without LPS (100 ng/ml) and DHA (0.1, 0.2, and 0.4 μM). *p < 0.05 vs. the model group. #*p* < 0.05 vs. the control group.

The protein levels of HIF‐1α, JAK3, p-JAK3, STAT3, and p-STAT3 were detected by Western blotting (Figure 4A). The expression of HIF‐1α, p-JAK3, and p-STAT3 in LPS-induced macrophages was significantly increased (Figures 4B,D,F). DHA at the doses of 0.2 and 0.4 μM decreased the LPS-evoked expression of HIF‐1α, p-JAK3, and p-STAT3 THP-1–derived macrophages. However, no difference in JAK3 and STAT3 levels was inspected in cells treated with or without LPS and DHA (Figures 4C,E).

## Discussion

Qinhao (the Chinese name of *Artemisia annua* L.) has been used as a traditional Chinese medicine for more than 2,000 years. Artemisinin, an extract of Qinhao, was discovered by Tu Youyou and has been known around the world for its potent treatment of malaria (Tu, 2011). In 1973, Tu Youyou’s group synthesized DHA by reducing artemisinin with sodium borohydride. The hydroxyl group in the structure of DHA made it more stable and more effective (Tu, 2011). Besides, drug metabolism study showed that DHA is also the primary active metabolite of artemisinin *in vivo* (Tu, 2011). Over the past decade, different indications for DHA have been explored. Numerous research studies showed that DHA may also be of therapeutic interest for some autoimmune inflammatory diseases, such as systemic lupus erythematosus (Li et al., 2006), psoriatic skin inflammation (Chen et al., 2020), and RA (Fan et al., 2018a; Fan et al., 2018b).

In this study, we treated CIA mice with DHA to assess its protective effects against inflammation and bone destruction in joints. The results showed that DHA significantly reduced the paw oedema and bone erosion in CIA mice. MTX is one of the DMARDs and has been the most commonly used in the clinic for the treatment of RA. In addition, it has become clear that the utilizing of MTX directly or indirectly affects the inflammatory response of synovial macrophages (Boutet et al., 2021). In this study, MTX was used as a positive control drug and showed potent anti-inflammatory properties in CIA mice. However, there was no significant difference in the efficacy of these two drugs. Besides, weight of the mice in the model group was significantly lower than that of the control group from day 35 to day 49, when the arthritis symptoms peaked in CIA mice. However, the body weight of mice in the control group, DHA group, and MTX group increased steadily during the whole experiment, and no difference was observed among these groups, which indicated that DHA or MTX improved the food intake in CIA mice. From another point of view, DHA and MTX improved the arthritis symptoms in CIA mice.

The serum levels of IL-1β and IL-6 in CIA mice were decreased after DHA treatment, which indicated that the inflammatory response was suppressed by DHA. Macrophages are dynamic cells that secrete large amounts of IL-1β and IL-6 and play a crucial role in the pathogenesis of synovium inflammation in RA (Yang et al., 2020; Boutet et al., 2021). Therefore, the LPS-induced inflammatory model in THP-1–derived macrophages was used to investigate the potential mechanism by which DHA inhibited inflammation in this study. The cytotoxicity of DHA on THP-1–derived macrophages was detected by CCK-8 assay, and three doses of DHA (0.1, 0.2, and 0.4 μM) with no cytotoxicity were used to perform the subsequent experiments.

IL-1β is typically used as a marker of inflammatory activation of macrophages due to its position at the apex of the pro-inflammatory cytokine cascade (Lopez-Castejon and Brough, 2011). Our results showed that the expression of IL-1β in these cells was significantly inhibited by DHA at the doses of 0.2 and 0.4 μM. So, the subsequent question is how does DHA decrease the IL-1β releasing in these cells?

NLRP3 inflammasome is a multi-protein complex that plays key roles in IL-1β maturation and secretion (He et al., 2016). Thereby, the expression of NLRP3 was evaluated in these cells. The results revealed that the protein level of NLRP3 in macrophages was apparently reduced by the treatment of DHA at the dose of 0.2 and 0.4 μM. This result was parallel to the IL-1β releasing.

Previous research indicated that the phosphorylated STAT3 (p-STAT3) facilitated histone H3 and H4 acetylation on Nlrp3 promoter, which promoted the expression of NLRP3 (Liu et al., 2018; Jiang et al., 2021b). STAT3 is one of the major signal transducers of JAKs and plays a role in inducing and maintaining an inflammatory microenvironment (Zare et al., 2015). STAT3 can be phosphorylated by JAK3. The elevated expression and dysfunction of STAT3 lead to the persistence of synovial inflammation in RA patients and animal arthritis models. A previous study in ulcerative colitis revealed that DHA impeded inflammation by inhibiting the JAK/STAT pathway (Jiang et al., 2021a). Nonetheless, it was not clear whether DHA inhibited the JAK/STAT pathway in THP-1–derived macrophages and, consequently, reduced NLRP3 expression. Subsequently, the expression of JAK3, p-JAK3, STAT3, and p-STAT3 was detected. Results showed that both of the p-JAK3 and p-STAT3 levels were reduced by the treatment of DHA at the doses of 0.1, 0.2, and 0.4 μM.

HIF-1α is a direct regulatory factor of the adaptive response to alterations in oxygen tension (Muz et al., 2009). With regard to RA, synovium in the inflamed joint is characterized by hypoxia, which leads to increased levels of the HIF-1α in macrophages within the synovium (Umar et al., 2021). Our results showed that LPS also induced the HIF-1α expression in macrophages, which was consistent with previous experiments (Williams et al., 2022). Some studies indicated that HIF-1α took part in the phosphorylation of STAT3, as the p-STAT3 protein was significantly inhibited with HIF-1α siRNA (Gao et al., 2015). Further research showed that the accumulation of HIF-1α triggered glycometabolic reprogramming in macrophages through which the phosphorylation of STAT3 was promoted. During the process of glycometabolic reprogramming, the expression of a key enzyme of glycolysis, named Pyruvate Kinase M2 (PFK2), is upregulated (Rodríguez-Prados et al., 2010). The dimeric PKM2 form acts as a protein kinase and phosphorylates STAT3 at Y705 (Damasceno et al., 2020). Besides, succinate, an intermediate of the tricarboxylic acid cycle, is accumulated during the HIF-1α–dependent glycometabolic reprogramming. The elevated expression of succinate promotes the phosphorylation of STAT3 *via* its receptor G protein-coupled receptor-91 (Mu et al., 2017). In this study, we noticed that the HIF-1α protein got significantly decreased under the treatment of DHA at the doses of 0.1, 0.2, and 0.4 μM. So far, our study supported the view that DHA reduced inflammatory response in macrophages by inhibiting NLRP3 dependent IL-1β release *via* the HIF‐1α and JAK3/STAT3 signaling pathway.

However, interaction between STAT3 and HIF-1α is still obscure. Some studies have indicated that HIF-1α is a downstream regulatory signal of STAT3. As showed by Wang *et al.* (Yang et al., 2022), the STAT3/HIF-1α/fascin-1 axis was involved in regulating RA fibroblast-like synoviocyte migration and invasion. Accordingly, the underlying mechanisms involved in the functional link between HIF-1α and STAT3 is yet to be elucidated. Besides, detection of the HIF‐1α and JAK3/STAT3 signaling pathway in CIA mice was lacking in this study. Therefore, we are expecting to conduct, in the future, more in-depth investigations to shed more light on the precise molecular regulatory mechanisms of DHA in RA treatment.

## Conclusion

In this study, DHA alleviated the inflammation and arthritic symptoms in CIA mice. Consistently, DHA impeded inflammatory response in THP-1 cells derived macrophages by decreasing the NLRP3 expression and IL-1β release. Additional research found that the HIF‐1α and JAK3/STAT3 signaling pathway involved in NLRP3 expression was also inhibited by DHA. Our data provided evidence supporting the potential of DHA in RA treatment and shed some light on the underlying mechanisms: DHA inhibited the NLRP3 expression through the HIF‐1α and JAK3/STAT3 signaling pathway in macrophages, and as a consequence, the following inflammatory response was prevented.

## Data Availability

The original contributions presented in the study are included in the article/Supplementary Material, further inquiries can be directed to the corresponding author.
